# Design and implementation of virtual fitting system based on gesture recognition and clothing transfer algorithm

**DOI:** 10.1038/s41598-022-21734-y

**Published:** 2022-11-01

**Authors:** Ying Wu, Hongbing Liu, Pengzhen Lu, Lihua Zhang, Fangjian Yuan

**Affiliations:** 1 Jiaxing Nanhu University, Jiaxing, 314001 Zhejiang Province China; 2 Hangzhou Dianzi University, Hangzhou, 310018 Zhejiang Province China; 3 Zhejiang University of Technology, Hangzhou, 310014 Zhejiang Province China

**Keywords:** Engineering, Mathematics and computing

## Abstract

Virtual fitting can bring fast and convenient fitting experience for people. The two core problems of virtual fitting system are human–computer interaction and clothing simulation. Interaction is an important factor in determining the fitting experience. The previous virtual fitting products usually use the mouse and keyboard interaction, and users rarely have a good sense of substitution and interaction. While, the method of using multiple cameras to take user images from different angles and then carry out posture recognition has the defect of low recognition accuracy. In view of clothing simulation and human–computer interaction of virtual fitting system, in order to achieve better customer immersion experience, this paper implemented a real-time interactive virtual fitting system based on Microsoft Kinect motion sensing device, and proposed a gesture determination algorithm based on finger recognition and an image transfer algorithm based on skeleton information matching. Using OpenNI development library and multi-threading technology, we have developed a motion-sensing capture module and a complete real-time virtual fitting system, and the system test results show that it has a good user experience.

## Introduction

Nowadays, the pace of life is gradually accelerating, a convenient and time-saving lifestyle has become a universal demand of people. According to a comparison survey of TOP10 categories of frequently purchased commodities among Chinese online shopping users in 2019, the sales volume of clothing products accounts for 56%, while 78% of online shopping customers have experience of returning or exchanging goods^1^. Clothing is a non-standardized commodity that is influenced by many factors, including size, color, style and even the feel and quality of the material. However, online clothing is difficult to meet many needs, in addition to the color is intuitive. Virtual fitting puts virtual clothes on the user through computer simulation technology. Users can watch the visual effect of the purchased clothes on themselves, and have a similar experience with on-site fitting, which can meet the needs of the public for other factors.

Online clothing display methods widely used by major clothing brands, such as Swedish brand H&M and Japanese brand Uniqlo, have launched virtual fitting services, which provide interesting user interaction, allowing users to set height, waist size and other measurements. However, the generated three-dimensional human body still cannot accurately simulate the customer's body shape. Some studies have shown that the same body size does not represent the same body shape^2^. In order to make the process of model creation more intelligent, some systems use multi-perspective images to complete automatic reconstruction of human model^3^. However, due to the computational complexity of multi-perspective reconstruction, the reconstruction process is often time-consuming and requires buyers to wait patiently. In addition to being used for online clothing buying and selling, some new virtual fitting systems put more emphasis on the user's virtual reality experience. Users can not only watch the image of virtual clothing, but also drive the actions of virtual avatars through their own posture, so as to obtain an immersive experience like "looking in the mirror"^4^. Yet now, the current virtual fitting system is still far from practical application. How to make the system more quickly and accurately identify the user's posture in dynamic changes and respond to the user's virtual operation is one of the key research issues in this kind of system. Therefore, this paper realizes an interactive virtual fitting system with dynamic fitting effect based on Kinect.

In order to achieve better customer immersion experience, this paper implemented a real-time interactive virtual fitting system based on Microsoft Kinect motion sensing device, and proposed a gesture determination algorithm based on finger recognition and an image transfer algorithm based on skeleton information matching. Therefore, gesture recognition and clothing transfer algorithm are the main topics of this study. Our goal is to use OpenNI development library and multi-threading technology to achieve the somatosensory acquisition module, accurately obtain the human skeleton and palm position and other information, to ensure the reliability of gesture judgment and posture matching based on this function. To achieve a complete real-time virtual fitting system and good user experience.

## Related work

### Virtual fitting method

For the research of virtual fitting, many technologies are not mature yet. In the past, the method often adopted was to enter user data to customize the virtual model image for rough static fitting^5^, which is widely used via web or App^6^. This method is relatively simple, but the fitting function is also very limited. Wu et al.^7^ made an attempt on virtual fitting based on graphics, adopted ellipsoidal hierarchical structure to approximate the human body model, simulated complex clothing patterns on this basis, and finally obtained the final visual design result through physical simulation. These implementations require a lot of equipment and are not interactive.

In recent years, many different image-based virtual try-on networks(VTON) have been proposed^8,9^, which use shape-context matching algorithms to twist cloth over the target figure without reference to a mannequin. These virtual fitting methods based on deep learning do not need any 3D information and can achieve good fitting results with good economy^10,11^. However, the generated images are prone to texture deformation and body parts loss, which may affect fitting results and can not interact.

With the popularity of deep acquisition devices such as Microsoft's Kinect and Intel's Creative Sensor, human body reconstruction methods based on deep data have attracted academic attention. Kinect integrates gestures, facial expressions, voice and other natural ways of human communication, becoming a new way of human–machine interaction, has been applied in a wide range of fields. Wang et al.^12^ constructed a virtual fitting system, using the depth camera to capture user posture and shape in a 3D environment with adaptive and constraint, and tracking and adjustment based on templates, and using data-driven generation of garment pleats to complete garment drawing and match with users. Ma et al.^13^ proposed an interactive virtual fitting system based on Kinect, which mainly studied the algorithm of reconstruction of human body model. Xue et al.^14^ proposed a kinect-based human fitting algorithm to achieve real-time performance. According to the body contour obtained from the scan, the human body model was reconstructed in real time. Aiming at the garment drawing problem, an ellipsoid tracking model was constructed to fit the human body model in real time. Shi et al.^15^ studied the correlation between human movement and garment deformation in view of the generation and disappearance of folds in garment animation. The relationship model between human motion and garment deformation is constructed to predict garment deformation accurately. Most of these methods use Kinect to take deep data information for reconstruction of human body model, but few of them can use gesture information to interact with the system, resulting in poor user immersion.

### Gesture interaction

Gesture interaction provides users with a natural way of human–computer interaction. Earlier approaches generally measured the motion data of finger joints with data gloves to realize gesture recognition. Among them, Lin^16^ proposed a real-time interaction method of 3D model based on data gloves and position tracker. Song et al.^17^ used mobile and wearable devices for motion tracking, and conducted a comprehensive study on inertial motion tracking methods. However, these devices are inconvenient and costly. Lu et al.^18^ used Leap Motion device to analyze the position and direction of each fingertip and identify gestures. Xu et al.^19^ proposed a gesture recognition method based on semi-supervised joint learning, using color segmentation algorithm to separate the contour of hand from the image and realize gesture recognition. Liang et al.^20^ presented a bared-hand depth perception method that was designed for improving interaction experience in ARASS and applied it to the augmented reality assembly system. Chen et al.^21^ proposed a robust real-time dynamic gesture recognition system for natural interaction with service robots in a dynamic environment. Tao et al.^22^ studied the influences of body posture, interaction distance and target size on the interaction between freehand and large screen.

### Clothing simulation

In addition to gesture interaction, clothing simulation is also a very important issue in the field of virtual fitting. How to represent and display clothes in the virtual fitting system directly determines the operation and maintenance cost and final effect of the fitting system. Martynov et al.^23^ studied the effects of color and pattern on visual perception of weight. Through a series of observations and experimental studies, they concluded that pure dark color significantly reduced weight perception compared with pure light color or horizontal stripe. Mir et al.^24^ proposed Pix2Surf, which digitally maps the texture of clothing images to the 3D surface of virtual clothing items. Yang et al.^25^ synthesized try-on images through three modules, which not only retained the characteristics of clothes, but also retained the identity details of people. Patel et al.^26^ proposed TailorNet, a data-driven clothing model based on posture, shape and style. This neural model can predict the three-dimensional deformation of clothing while preserving the details of wrinkles. Han et al.^27^ proposed ClothFlow for attitude guidance synthesis and image-based virtual fitting. WANG et al.^28^ proposed FVTN to learn garment deformation by using stream-based methods. Adikari et al.^29^ adopted a simple 2D texture for the representation of clothing, and the display method of clothing was to attach the texture to the image of people according to the information of human skeleton, and adjust it through image transformation. However, the existing motion sensing virtual fitting system cannot well represent the physical characteristics of clothing, such as drape, fold, elasticity, etc.^30^. It is difficult to give users a good sense of substitution and mutual sensing and identify the defects of accuracy. Aiming to provide users with a good sense of substitution, mutual perception and recognition accuracy, this paper adopts a new garment representation and synthesis technology, and realizes a complete interactive 3D virtual fitting system based on Kinect motion-sensing equipment. The system mainly involves three dimensional visualization, motion sensing interaction, gesture recognition and clothing simulation.

## Methods

**Figure 1:**
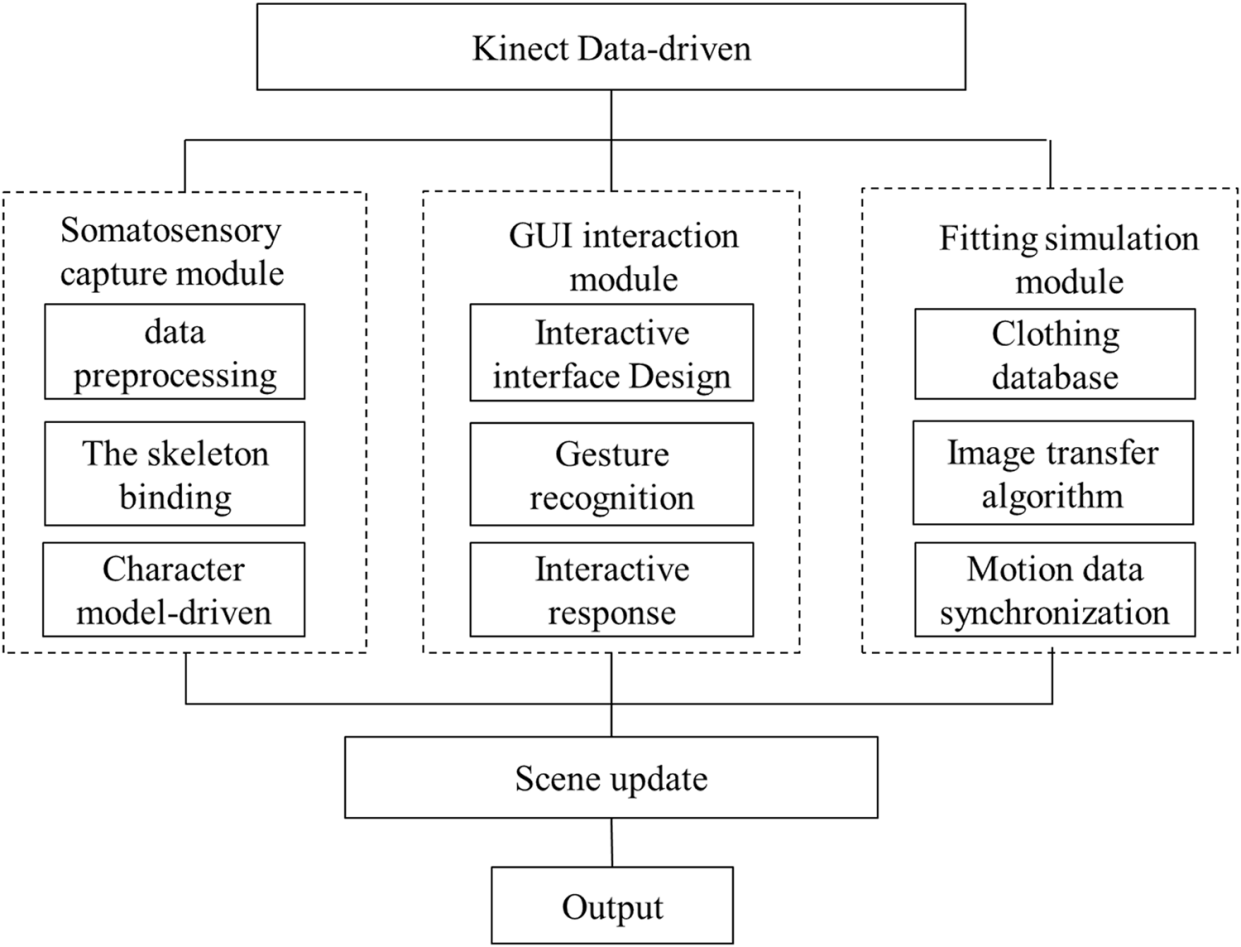
System flow conception.

The purpose of this study is to achieve a virtual fitting system that allows users to control the Graphics User Interface (GUI) of the software through their body and gestures, so as to achieve an immersive experience like "looking in the mirror". Kinect somatosensory interaction device is used for external devices, and all 3d related development and implementation are based on OpenSceneGraph (OSG) engine. The system structure is shown in Fig. 1. Among them, the somatosensory capture module is responsible for using OpenNI development library to call Kinect somatosensory device and obtain color image and depth image through multi-threaded programming, and on this basis, identify the skeleton, palm and other information. The GUI interaction module realizes a human–computer interaction interface based on gesture recognition. The system uses this interface to display clothing items, and users operate the interface menu and select items through gestures. The fitting simulation module is mainly responsible for retrieving the preprocessed garment images from the database, matching the target image most suitable for the current posture, and transferring it to the current image to generate the final fitting results.

### Gesture determination algorithm based on fingertip recognition

The interactive gestures of users in the GUI interactive module in this paper are mainly divided into two types: moving hands and grabbing actions. The hand detection and tracking are realized by the relevant interfaces provided by OpenNI, while grasping is the most common and convenient gesture. Due to its complexity and the shortage of Kinect hardware, there is no relevant interface provided in the library. We propose a gesture determination algorithm based on fingertip recognition to realize gesture interaction design.

The main ideas and flow of gesture determination algorithm based on fingertip recognition are as follows: Firstly, the nearest neighbor domain method is adopted to divide hands from the depth map captured by Kinect. Then the hand contour is extracted from the binarization image. The barycenter method was used to optimize the traditional Graham scanning algorithm^31^, and the convex envelope points, also known as fingertip candidate points, were calculated from the previously obtained contour points. The contour analysis method based on curvature feature is further used to identify the fingertip^32^.According to the number of fingertips, and combined with the area ratio method to determine grasping gesture.

In order to achieve the fingertips identification features, first we need to pass the device body feeling device to capture the depth of the current frame corresponding to the image, on the depth of it is processed by Gaussian filtering, whose function is to weighted average of the whole image, so that the value of each pixel is weighted and averaged by the value of pixels in the surrounding area, so as to reduce the interference of noise. Two-dimensional Gaussian filtering is shown in Formula (1).

1 $$g(x,y) = \frac{1}{{2\pi \sigma^{2} }} \cdot e^{{ - \frac{{x^{2} + y^{2} }}{{2\sigma^{2} }}}}$$

Secondly, separate the hand from the depth image. The depth map captured by Kinect shows how far an object is from the camera in grayscale. Agree to extend the hand to the front of the body during recognition, so that the gray value of the hand in the depth map will be smaller than that of the back body and the surrounding environment. Then, according to the palm depth value obtained in advance, a threshold T is set in the z-axis direction, the points plus or minus T within the range of the palm depth are extracted and binarized according to Formula (2).

2 $$hand(x,y) = \left\{ {\begin{array}{*{20}l} {0,} \hfill & {depth(x,y) \le hc.z - T} \hfill \\ {0,} \hfill & {depth(x,y) \ge hc.z + T} \hfill \\ {255,} \hfill & {Otherwise} \hfill \\ \end{array} } \right.$$

In the above formula, *hc.z* is the depth value of palm, T is the thickness threshold of palm, depth(x, y) is the depth value of the point (x, y) in the original depth image captured by Kinect. Hand (x, y) is the value of the point (x, y) in the separated hand depth image after binarization.

**Figure 2:**
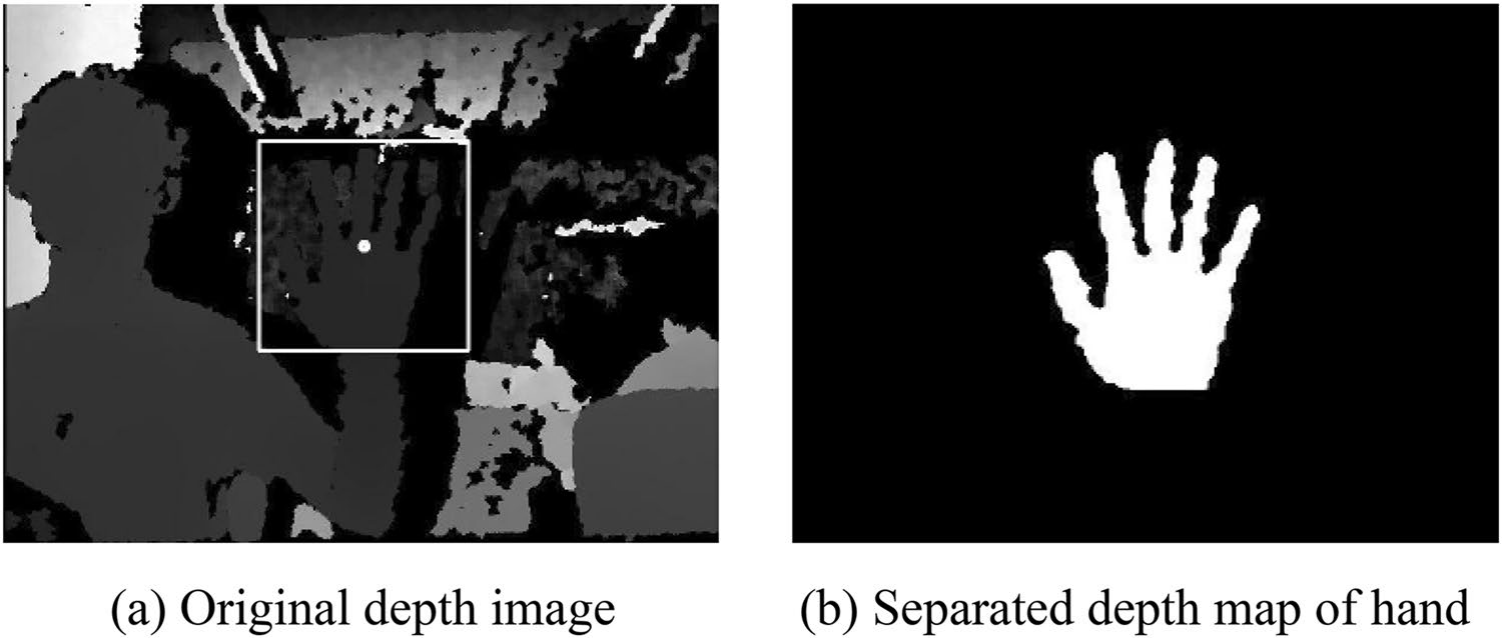
Hand segmentation effect.

In order to facilitate viewing the results, the part of the hand is highlighted with a white rectangle. The binarization hand depth image was separated by the nearest neighbor domain method. The hand segmentation effect diagram obtained in the experiment and the original depth image captured by Kinect are shown in Fig. 2.

After separating the binary image of hand from the depth of the depth map, the hand contour needs to be extracted from the binary image to facilitate the calculation of convex hull. In this paper, a relatively simple method is adopted to obtain the hand contour: The binarization image was traversed line by line, and a variable PreColor was used to mark the color value of the previous pixel point. The color value of the current pixel point was compared with PreColor each time. If the color value was different, that is, the color changed, which indicated that the current point was a point on the Contour, and the point was saved to {Contour} set. After traversing the whole image, an array {Contour} containing all Contour points was obtained, which was used as the input of the convex hull calculation in the next step. The fingertip recognition algorithm proposed in this paper is based on convex envelope calculation.

The motion sensing fitting system in this paper is based on gesture interaction, so the definition and judgment of gesture is very important. Gestures can be divided into many types, but as for interaction methods, it is not good to be too detailed or too complex, which will cause trouble for users to operate, and the efficiency and accuracy of complex gesture recognition will be greatly reduced. Therefore, in line with the idea of simplicity and good interactivity, only "grab" gesture is defined in this system in response to the "OK" command.

The GUI of this system is all "button" type. In common operating systems that use mouse interaction, the response of buttons is through the mouse "click" command. However, in somatosensory interaction, although OpenNI provides the "press" gesture interface similar to the "click" command, the effect is always not ideal and the operation is not convenient enough. In contrast, "grab" action as the most basic hand action, it is convenient to complete, so this paper defines "grab" action to achieve the effect of "click". Gesture judgment in this paper also revolves around "grab" gesture.

"Grasping" action can be divided into "open" and "holding up" two continuous states. Each state can be judged by tipsN, the number of fingertips obtained in the previous step. In view of the complexity of hand shape, interference of convex envelope or fingertip loss may occur occasionally during fingertip recognition. In this case, the palm state can be determined by setting a threshold T.

However, it is not stable to judge the palm state only by tipsN. In order to obtain better interactive effect, this paper adds the condition of area ratio on the basis of fingertip number to ensure the correctness and stability of gesture recognition. OpenCV provides the "contourArea" function to calculate the area of a given contour. Define the area of the palm as S_hand_ and the area of the convex hull as S_Contour_. The proportion of the two is:

3 $$scale = \frac{{S_{hand} }}{{S_{Contour} }}$$

When the palm is in the open state, there are gaps between the convex edge and fingers, so:

4 $$S_{hand} < S_{Contour}$$

On the contrary, when the palm is in the holding state, the area of the palm is still smaller than the convex hull area, but the difference between the two is not significant, so the threshold is set as S and compared with scale. Set palm status as Status, then:

5 $$Status = \left\{ {\begin{array}{*{20}l} {grabbed,} \hfill & {N_{tips} \le T,\quad scale \ge S} \hfill \\ {released,} \hfill & {otherwise} \hfill \\ \end{array} } \right.$$

The palm state of a single frame can be obtained through the above formula. "Grab" gesture is an action involving the palm state of several consecutive frames within a period of time. In this paper, a buffer pool of size N is set up to save the palm state of the previous N frames. The state of the N frames is analyzed, take the large number of states as the Status_pre_ of the previous N frames, and then combine the Status_cur_ of the current frame to determine whether "grab" gesture occurs:

6 $$Gesture = \left\{ {\begin{array}{*{20}l} {occurred,} \hfill & {Status_{cur} = grabbed,\,Status_{pre} = released} \hfill \\ {otherGes,} \hfill & {otherwise} \hfill \\ \end{array} } \right.$$

"Grab" action occurs, hand towards will have a variety of situations, this paper in view of the fingers up and down, the left and the right four kinds of circumstances, collected 10 subjects of gestures, each subject is done 5 times for each position "grab" action. Finally, the results of 50 grasping experiments are obtained. The recognition times and preparation rate of "grasping" action in each case are shown in Table 1.

**Table 1:** Grasping gesture recognition rate in four cases.

Hand towards	Upwards	Downwards	Towards the left	Towards the right
Identification times/times	49	41	43	45
Accuracy rate/%	98	82	86	90

Experimental results show that under each orientation of the hand, the recognition rate of "grasping" action is more than 80%. For the recognition failure, there are two main reasons: First, the depth map returned by Kinect somatosensory device will sometimes be broken, resulting in the recognition error of convex hull, and finally the recognition result is wrong; Secondly, wrist deflection occurs in the two cases of downward and left orientation, which leads to the sharp Angle of the extracted hand contour at the wrist and is difficult to filter. Therefore, it is occasionally misjudged as fingertip by the fingertip recognition algorithm, resulting in a relatively low accuracy of gesture judgment in these two cases. In general, the gesture recognition algorithm presented in this paper has high recognition rate and strong robustness.

### Clothing image matching and transfer algorithm

Traditional fitting systems tend to study the simulation algorithm of clothes, and simulate the physical properties of clothes through 2D image transformation or 3D modeling and animation simulation. However, due to the complexity of physical properties of clothing, the simulation results of these methods are lacking in authenticity and texture.

**Figure 3:**
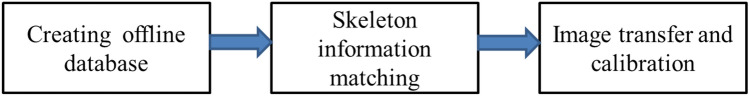
Flow of clothing transfer algorithm.

Stefan et al. published a paper named "Image-based Clothes Transfer"^33^, in which they proposed an image-based clothing Transfer algorithm, which cleverly avoided such thorny problems as clothing material simulation and clothing 3D model reconstruction. Instead, the offline clothing image database is created and the method of contour feature matching is used to retrieve the clothing image most suitable for the current contour from the number algorithm analysis database, and then the clothing fitting is carried out. This algorithm has strong practicability, convenient operation, and can well reflect the texture of clothing, wrinkles and other physical characteristics that are difficult to be simulated by algorithm. This paper improved the algorithm based on Stefan et al., combined the motion sensing technology into the algorithm, adopted the motion sensing information as the key word of matching, and proposed the clothing transfer algorithm based on Kinect bone information. The process of the whole algorithm is shown in Fig. 3.

#### Offline database creation

The corresponding database of this paper refers to the collection of clothing model image data. The system records a series of model images for each garment commodity, and conducts offline matting processing on the obtained images to separate the garment from each frame of image.

**Figure 4:**
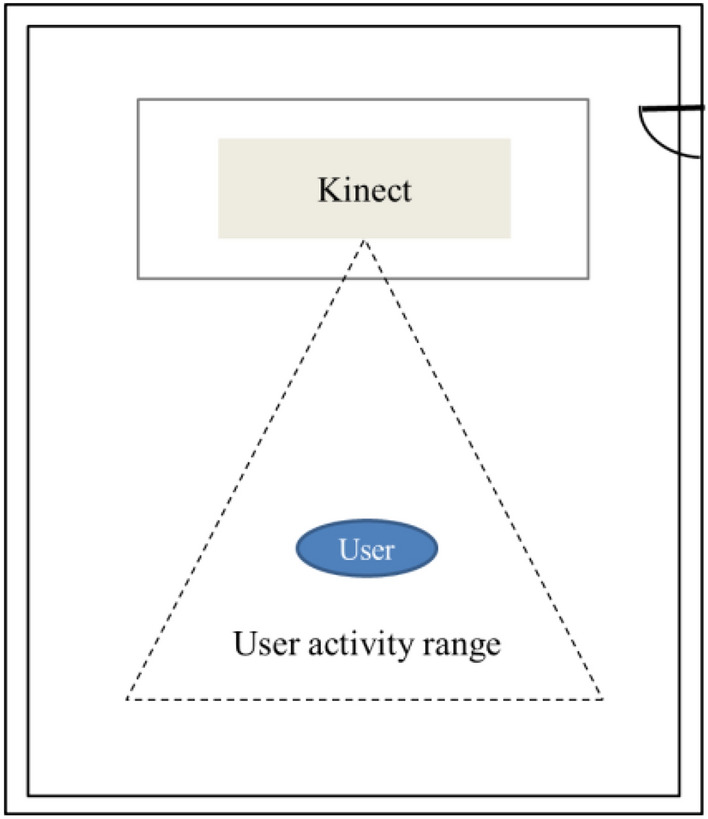
Top view of the room for image collection.

The acquisition device in this paper is a Kinect camera, which is placed above the display. According to the restrictions such as the nearest and furthest viewing distances and the lens opening Angle, the activity range of the model is obtained, as shown in the area surrounded by dotted lines in Fig. 4. This image capture device does not require a closed room or many cameras, making the database creation process more flexible.

In the process of image collection, in addition to saving the image of each frame, it is also necessary to save the skeletal position information of the model at the current moment. The OpenNI development library can be used to identify the information of 24 skeletal nodes of human body, and these joint information is used as the keywords for matching in this paper. In this paper, human contour information is avoided as the key word of the matching algorithm. Therefore, in the process of post-processing, it is no longer necessary to extract the contour and silhouette information of human body, but only to separate the part of clothing from the image. In order to get better results, we adopts the way of artificial matting, using Photoshop software for image matting and separation.

#### Clothing image transfer based on skeleton information matching

Clothing image transfer algorithm is a way to solve the problem of virtual fitting. It avoids the problems of physical property simulation and 3D clothing modeling in traditional virtual fitting implementation methods. Instead, the garment image closest to the current frame is retrieved from the pre-processed database by matching keyword information, and then the garment image is synthesized into the current character image through image transformation and calibration, so as to obtain the final result of virtual fitting.

In Stefan et al.'s paper^32^, image matching uses feature vectors composed of features extracted from contours as matching keywords. This method is highly dependent on the accuracy of contour extraction. Although each image in offline database can be manually extracted and stored locally, for the image corresponding to the current frame captured in real time, the contour can only be extracted by algorithm. Because of the complexity of image, automatic contour extraction is difficult to achieve a very ideal effect. In addition, the feature extraction method adopted in this paper only discretely extracts a specific number of feature points from the contour information, and the attitude information described by these feature points is very limited, so the accuracy of matching results obtained by feature vectors composed of these feature points is also very limited.

**Figure 5:**
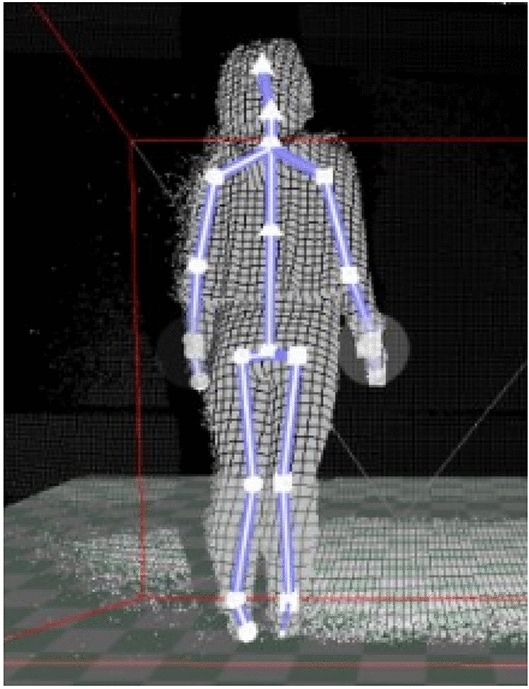
Human skeleton captured by Kinect.

We have improved the matching keywords. In combination with the characteristics of Kinect, the feature vector composed of human skeleton information recognized and tracked by OpenNI is used as the matching keywords. The human skeleton captured in real time by Kinect is shown in Fig. 5.

OpenNI can recognize and return the 3D coordinate values of 24 skeletal nodes of human body, and these 24 groups of values can constitute a feature vector $V_{ST}$, which can be expressed as follows:

7 $$V_{ST} = [ST_{1} ,ST_{2} , \ldots ,ST_{24} ]$$

$ST_{i}$ of each dimension is a three-dimensional coordinate:

8 $$ST_{i} = [X_{i} ,Y_{i} ,Z_{i} ]\quad i \in [1,24]$$

**Figure 6:**
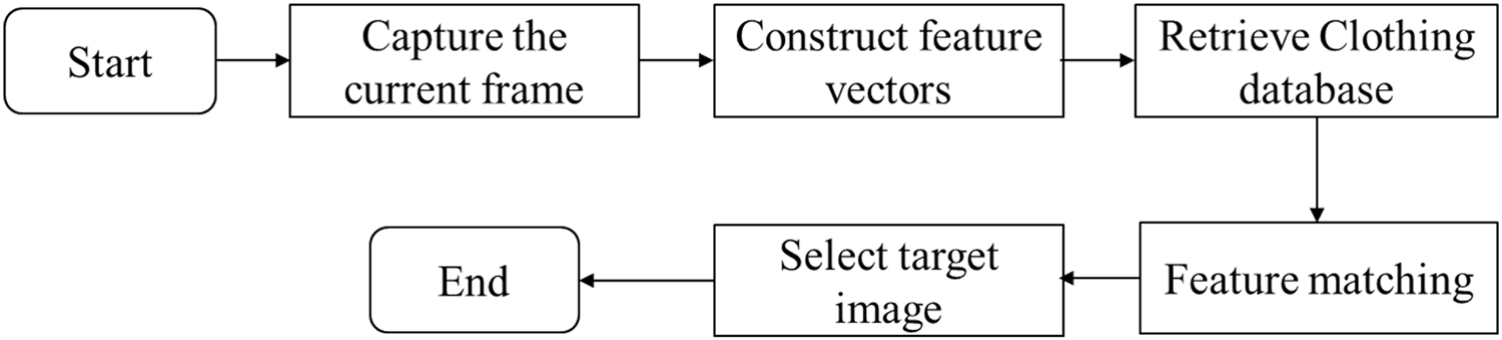
Clothing image matching algorithm.

The process of the transfer algorithm is basically the same as the former, as shown in Fig. 6, which is mainly divided into the following steps: capture the image corresponding to the current frame, obtain the bone position information through OpenNI, and construct the feature vector $ST_{i}$; Retrieval database and feature matching; Get the target image and fit it with the current frame.

In the whole process, database retrieval is the core stage. In this step, the system needs to search the database, match the current feature vector with the images in the database, calculate an energy value E for each pre-processed image in the database, and select the target image by controlling and limiting the energy E. Stefan et al. used the following formula to define energy:

9 $$E = D(l_{i} ,i)$$

where, $i$ and $l_{i}$ are n-dimensional vectors, and n is the number of contour feature points after principal component analysis. $i$ represents the feature vector of the current frame, and $l_{i}$ represents the feature vector of the current image in the database. $D(l_{i} ,i)$ is the Euclidean distance between $i$ and $l_{i}$. Let $i$ and $l_{i}$ be expressed as follows:

10 $$i = [i_{1} ,i_{2} , \ldots ,i_{n} ]$$

11 $$l_{i} = [l_{i1} ,l_{i2} , \ldots ,l_{in} ]$$

the Euclidean distance of the two is:

12 $$D(l,i) = \sqrt {(l_{i1} - i_{1} )^{2} + (l_{i2} - i_{2} )^{2} + \cdots + (l_{in} - i_{n} )^{2} } = \sqrt {\sum\limits_{j = 1}^{n} {(l_{ij} - i_{j} )^{2} } }$$

Using Euclidean distance as energy calculation formula can achieve the matching function well, but it can not reflect the continuity between adjacent frames well. Because in the process of fitting, there is a certain continuity of actions in a short period of time, and if only according to the Euclidean distance to identify, the image may not be adjacent to the previous frame retrieval results. This inevitably leads to the phenomenon of "flickering" or "mutation". If two adjacent frames match adjacent images in the database, the phenomenon of clothing "mutation" will be greatly improved. Therefore, we improved the energy calculation formula, and redefined the formula as follows:

13 $$E = D(l_{i} ,i) + \lambda C(l_{i - 1} ,l_{i} )$$

The meanings of $i$, $l_{i}$ and $D(l_{i} ,i)$ are still as mentioned above. We add a coherence judgment function $C(l_{i - 1} ,l_{i} )$ on the basis of Euclidean distance, which is defined as follows:

14 $$C(l_{i - 1} ,l_{i} ) = \left\{ {\begin{array}{*{20}l} {0,} \hfill & {l_{i - 1} \,and\,l_{i} \,are\,adjacent\,frames} \hfill \\ {1,} \hfill & {l_{i - 1} \,and\,l_{i} \,are\,not\,adjacent\,frames} \hfill \\ \end{array} } \right.$$

$\lambda$ is a threshold value, which is determined by experiment (the experimental value here is 100). The significance of $C(l_{i - 1} ,l_{i} )$ is that, by determining the relationship between the retrieval result of the previous frame $l_{i - 1}$ and the current database image $l_{i}$, the energy value of the adjacent frame is smaller than that of the non-adjacent frame, so that it has a greater chance to become the target image, and the method is to add a threshold value to the non-adjacent frame.

Experiments show that this improved image matching algorithm has better smoothness and continuity, and the keyword matching based on bone information in this paper has less dimension than that based on contour feature points, but it can better represent the human body posture information, so it has a good improvement in efficiency and accuracy.

#### Transfer and calibration of clothing images

After the above feature matching stage, a frame of target image is retrieved from the database. The collected image needs to be calibrated proportionally before it can be fitted into the current image. We use shoulder width to calculate the scale, and the formula is as follows:

15 $$scale = \frac{{X_{RS} - X_{LS} }}{{X_{RS}^{\prime } - X_{LS}^{\prime } }}$$

$X_{RS}$ and $X_{LS}$ represent the abscissa of the left and right shoulder joints in the current image respectively, $X_{RS}^{\prime }$ and $X_{LS}^{\prime }$ represent the abscissa of the left and right shoulder joints in the target image retrieved from the database. Scale is used to scale the target image, and then the target image is fitted to the current image by referring to the coordinates of the torso nodes in the current image.

Assuming that the coordinate axes of the two images take the XOY coordinate system as the standard coordinate system, there are several cases:

1. When $X_{RS}$ = $X_{LS}$ and $X_{RS}^{\prime }$ = $X_{LS}^{\prime }$, scale = 1, in this case, the database image obtained can be directly fitted with the current image obtained without scaling.
2. When the coordinates of the left and right shoulder joints of the target image are shifted to the right or left at the same time relative to the current image, the two images still cannot be fitted even if scale is 1.Therefore, we should also define the coordinate of the midpoint of the left and right shoulder joint line X = 0, where
  16 $$X = X_{RS}^{\prime } + X_{LS}^{\prime }$$
  If $X{ = }0$, it means that the two images just fit, and if $X > 0$, move the target image to the left by $X/2$ units, otherwise, do the opposite.
3. When $X_{RS}$ = $X_{RS}^{\prime }$ and $X_{LS}$ = $X_{LS}^{\prime }$, the image in the database needs to be scaled according to the value of scale to fit into the current image.

After the above steps, the clothing image transfer algorithm based on bone information matching can be realized. By improving the algorithm based on contour feature matching, this algorithm has better operation efficiency, accuracy and continuity. The specific experimental results will be shown in detail in Sect. 5.

### Ethics approval

This article does not contain any studies with human participants and/or animals performed by any of the authors. The formal consent is not required in this study.

### Statement for portrait

Our study complies with relevant institutional, national, and international guidelines and legislation. The photos in this paper were collected with the permission and use of the photo itself (the person in the photo (Figs. 10, 11, 12) is the author Wu Ying). We confirming that informed consent were obtained from all subjects and/or their legal guardian(s).

## Design and implementation of virtual fitting system

**Figure 7:**
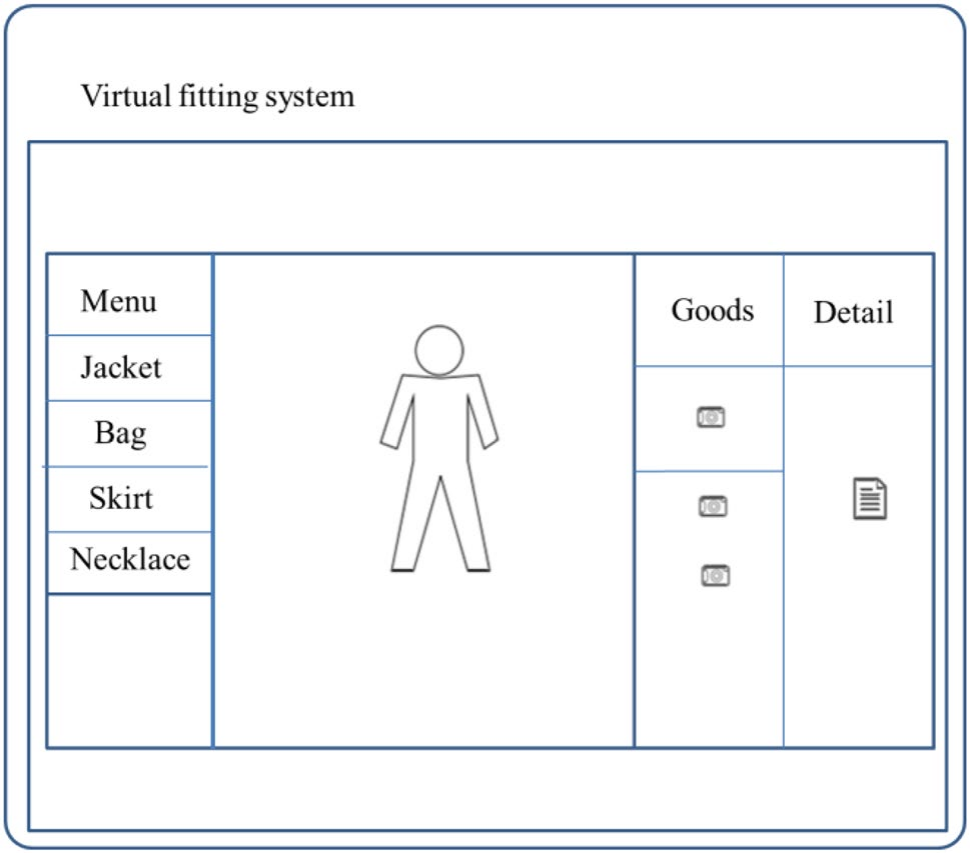
GUI interface layout design.

The GUI window layout design of this system is shown in Fig. 7. The whole interface is composed of two viewports. Viewport 1 occupies the largest space and is composed of three parts: the "menu" list on the left, the "items" list on the right and the user image area. The user image area is full of viewport 1. Due to the size of the user image captured by kinect is 640 × 480, the length to width ratio of viewport 1 is also restricted to 4:3 to avoid proportional distortion. The left and right lists are written and implemented based on OSG 3D engine. Each list item is represented by a quadrilateral, and the transparent blending option is turned on, so that the menu area is still visible when the user's palm enters.

Each list item in the "Menu" list represents a kind of clothing or jewelry. The user moves the palm to enter the list area, and the system judges the selected list item according to the recognized palm position and displays it as pressed state. When the user selects an item of clothing on the left, a list of items and a "cancel" button next to the list will pop up on the right. Each item in the list is an image of an item of clothing related to the selected category. Similarly, users can continue to choose their favorite clothes by hand, and then after the processing of the fitting module, the clothes will be synthesized into the current image and displayed in real time. Users can cancel the current fitting effect by clicking the "Cancel" button at the top right. Both the left and right lists have the function of scrolling, that is, when the display space is limited but there are many list items, the list items will slide in the opposite direction according to the user's palm movement, and the sliding speed is proportional to the speed of the user's palm movement. In addition, all choices are determined by the user's "grab" gesture, meaning that the corresponding option will be selected or responded to only when the user "grabs" action. Viewport 2 is the "Details" area on the far right, which displays detailed information about the item selected by the user, such as price, material, size, etc.

### Interactive Menu Response and update

**Figure 8:**
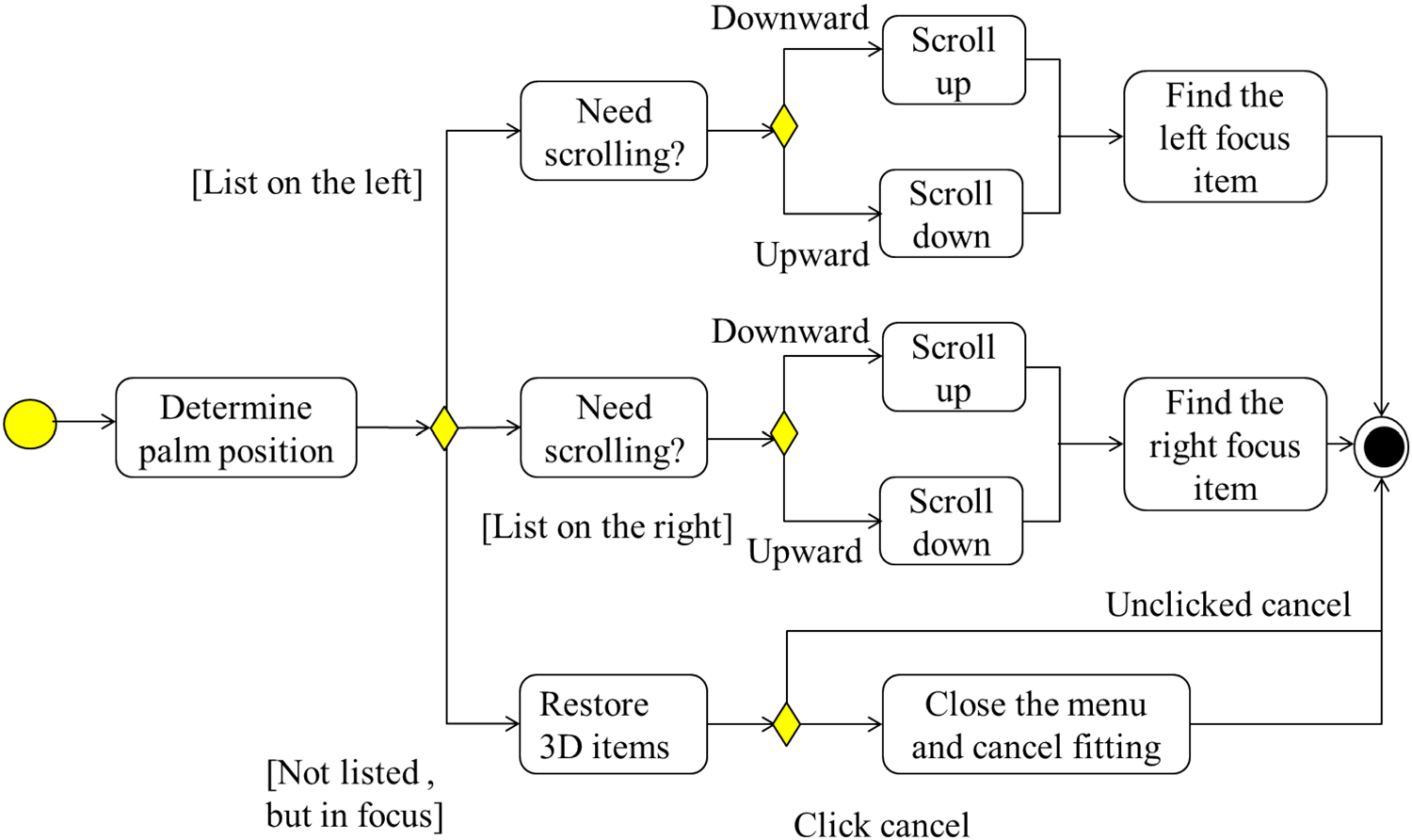
Activity diagram of interactive response module.

In GUI systems, the core is menu response and update operations. According to the main program flow, after the depth image is captured by Kinect device, algorithm processing such as palm recognition, fingertip recognition and gesture determination will be carried out. The gesture decision will result in a bool variable indicating whether the fetching action is currently taking place. This bool variable and the position of the current palm will be passed as parameters to the GUI module's Update function, which is responsible for processing the interactive response and updating the GUI's scene tree status. The Update function first calls the ProcessMenu function to handle the interactive response, retrieving the currently selected control and updating the coordinate position of the corresponding quadrilateral for each list item if the list slides. Then update the contents of the "Items" list on the right and the description information in viewport 2 according to the change of the focus of the list item. After processing, the Update function returns an object to the main program that contains the current GUI system status. The activity diagram for the ProcessMenu module is shown in Fig. 8.

### Somatosensory module design

**Figure 9:**
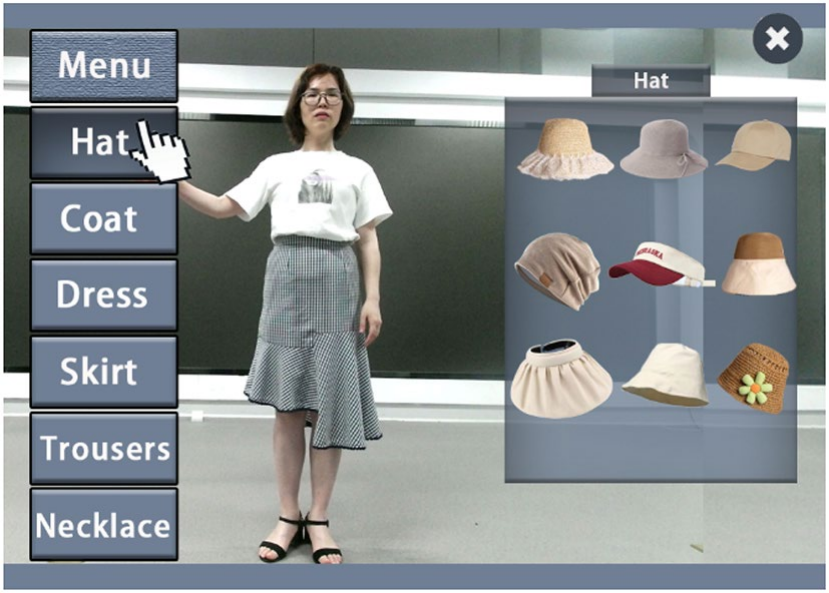
Flow chart of shared somatosensory data algorithm.

In the somatosensory capture module, Tracker class is encapsulated to achieve the acquisition of depth images, bone information and other functions. The Tracker class inherits from thread and shares the motion-sensing data captured by kinect with OpenNI internal threads through the use of multi-thread synchronization mechanism. The RUN function is the core part of the Tracker class. The logical flow of the algorithm in the RUN function is shown in Fig. 9.

### Design of fitting module

The clothing items involved in this system can be generally divided into two categories. One is items like handbags, which are represented by 3D models. The other is all kinds of clothing, which is simulated by the above image transfer algorithm based on bone information matching.

## System test and effect analysis

When the software starts running, the user needs to stand at a certain distance (2–3.5 m) from the camera and make an initial pose with both hands up, waiting for the skeleton to be generated. As the skeleton formation is complete, palm recognition is performed by waving hands. When it is recognized, the cursor will appear at the palm and move with the palm, this system interacts with the menu by moving the hand.

**Figure 10:**
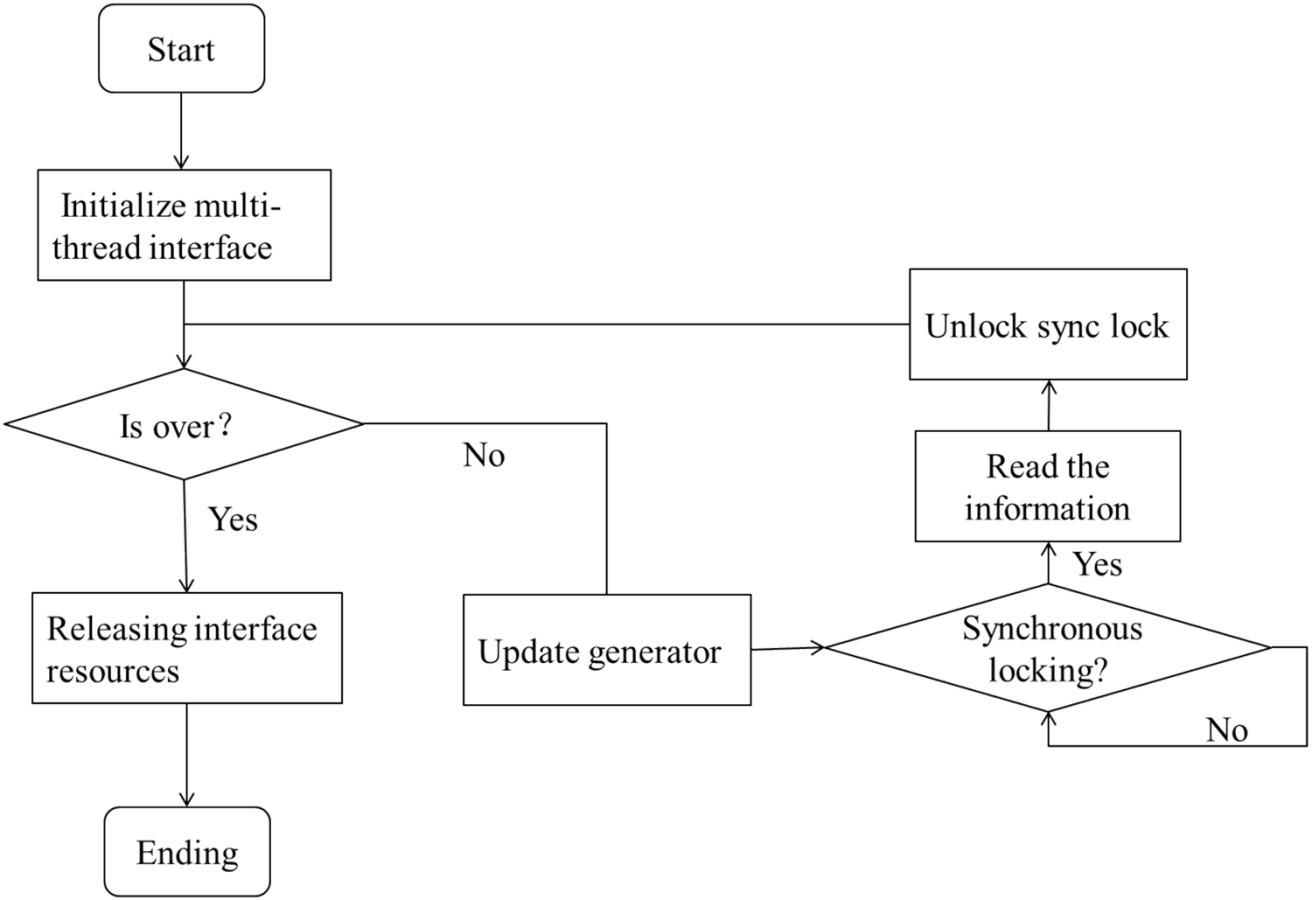
Select the left menu.

The menu system in this article consists of two parts: the menu list on the left of viewport 1 and the items list on the right of viewport 1. "Menu" list will be displayed after system initialization, and its content is all clothing categories supported by the system. The user uses gesture to select categories, and through gesture determination algorithm, the user can complete the selection of a list item with "grab" action. The process is as follows: place your open hand over the list item you want to select, and then hold it in your hand so that the cursor changes from a clipper to a fist. When a category is selected, the Items list and cancel button pop up to the right of viewport 1, as shown in Fig. 10. The menu of the system supports the scrolling function. When the list items are too many to display all, the list will be reversed according to the movement direction of the user's hand, and the scrolling speed is proportional to the movement speed of the user's hand, so as to facilitate the user's selection.

The selection of the items list on the right is the same as on the left, when the user selects a list item on the right by "grabbing", the system will calculate the fitting effect by using the fitting module.

For complex garments, such as coats and skirts, we adopts the above clothing image transfer technology based on skeleton information matching for simulation. Under the condition that the posture information of the pre-processed clothing image database is sufficient, the image transfer algorithm can achieve ideal results. The system can match the nearest image data from the database according to the current skeleton attitude information, and combine the skeleton information to scale and fit.

**Figure 11:**
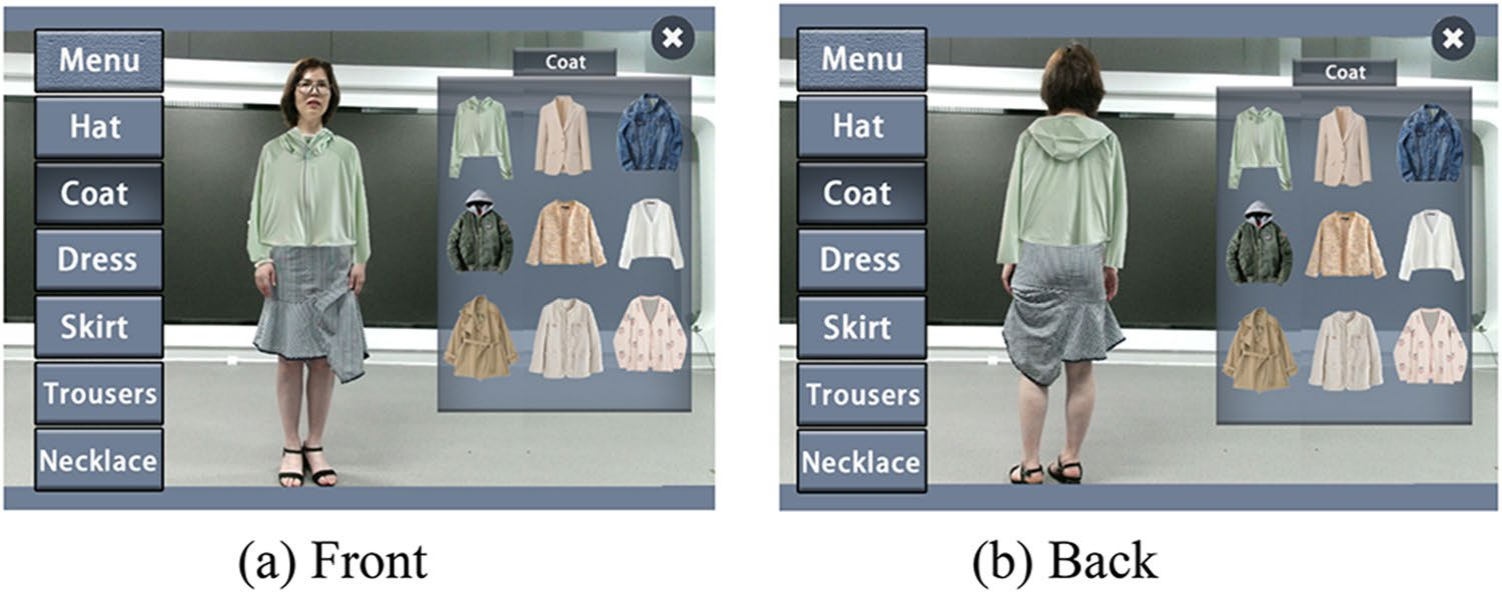
Virtual fitting effect of "Coat".

First, select the "coat" list item in the left list, and then select a coat garment in the "Items" list item on the right pop-up. At this time, the program will match the image of the coat retrieved from the database that is closest to the current posture to the original image. As shown in Fig. 11, the coat in the figure is the image transferred by the algorithm. Due to the high fit with the current action and the fine matting processing in the later stage, the final synthesis effect is very good, with only a slight deviation which is within the acceptable range. After selecting a certain dress, the user can take a common pose, and the system searches the database according to the identified skeleton information to match the results as appropriate as possible.

**Figure 12:**
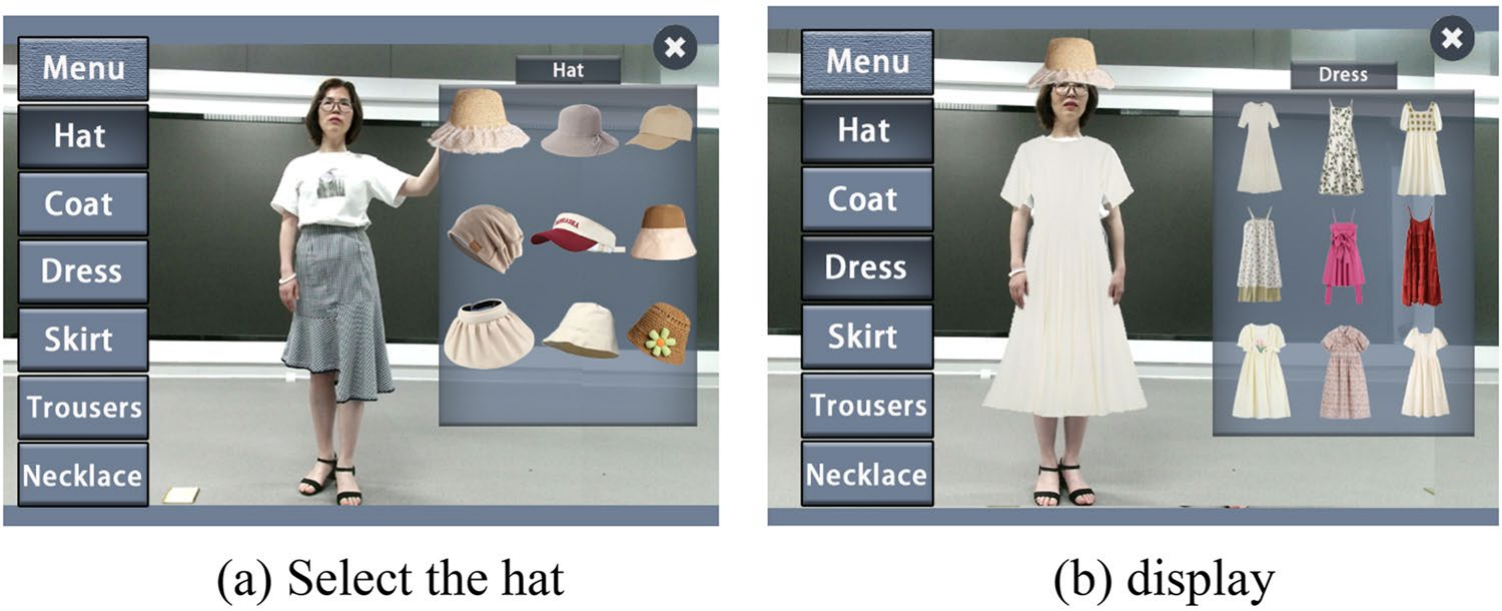
Mixed display effect of hat and dress.

The experimental results show that for more than half the input images, the deviation of the matching results can be within the acceptable range. However, in view of the complexity of the establishment of clothing image database, it is very difficult to achieve coverage of the vast majority of poses, so deviations such as those in Fig. 12 are inevitable. In this case, if the user is more concerned about the wearing effect of the current posture, he can adjust his own movements appropriately to adapt to the matching result, which usually achieves ideal results in the end.

Generally, after the user finishes the "fitting" experience of a certain category of clothing, he can click the "Cancel" button to clear the current fitting effect, and then experience the new category or exit the system. In addition, the system also supports a mixture of 3D objects and 2D clothing display effects. Operation is also very simple, after selecting a certain clothing, do not click the "cancel" button, but continue to select other clothing, you can achieve the effect of mixed display. Figure 12 shows a mixture of "hat" and "dress". Based on the above modules, a complete real-time virtual fitting system is realized, and the system testing effect is verified, with good user experience.

## Conclusion

For virtual fitting system, the two core problems are human–computer interaction and fitting simulation method. Interaction is a very important factor in determining the fitting experience. Many virtual fitting room products in the past are difficult to give users a good sense of substitution and interaction. Based on the shortcomings of existing studies, in order to realize customers' immersive virtual fitting experience, we provide some solutions. The main conclusions are as follows:

1. We use Kinect somatosensory device to realize interaction. Based on OpenNI development library and multi-thread technology, we realize the somatosensory capture module, which can accurately obtain information such as human skeleton and palm position, ensuring the reliability of gesture judgment and posture matching.
2. We propose a gesture determination algorithm based on fingertip recognition, and encapsulates a gesture-based human–computer interaction module. The man–machine interaction interface based on this algorithm enables users to operate the system with simple and convenient gestures, which has a good interaction effect.
3. The fitting simulation technology based on skeleton information matching image transfer algorithm is realized. The skeleton information collected by the motion sensing equipment is used as the matching keyword. Compared with the contour information, our method has higher accuracy and faster computing speed, and the requirements of hardware equipment are greatly reduced. In addition, this paper improves the matching algorithm by adding continuity judgment information to the original energy calculation formula, and the new matching algorithm has stronger continuity.

## Data Availability

The datasets generated during and/or analyzed during the current study are available from the corresponding author on reasonable request.
